# Analysis of vulnerabilities and negatives outcomes of tuberculosis treatment in the Municipality of São Paulo, 2019 and 2021

**DOI:** 10.1590/0037-8682-0445-2024

**Published:** 2025-10-20

**Authors:** Rodrigo Contrera do Rio, Maria Josefa Penón Rujula, Rita Barradas Barata

**Affiliations:** 1 Santa Casa de São Paulo, Faculdade de Ciências Médicas, Departamento de Clínica Médica, São Paulo, SP, Brasil.; 2 Santa Casa de São Paulo, Faculdade de Ciências Médicas, Departamento de Saúde Coletiva, São Paulo, SP, Brasil.

**Keywords:** Social vulnerability, Tuberculosis, Descriptive epidemiology, Unfavorable outcomes

## Abstract

**Background:**

To study the unfavorable outcomes of tuberculosis treatment, presenting individual, social, and program vulnerabilities in homeless, HIV-infected individuals and individuals living in areas of high social vulnerability in the city of São Paulo.

**Methods:**

Cases were reported, confirmed, and monitored using the tuberculosis control program in the city of São Paulo. Social vulnerability was assessed using the São Paulo Social Vulnerability Index (SVI). Individuals analyzed in the study with more than one vulnerability condition were allocated to more than one vulnerability group, and the outcomes were analyzed separately. The reference category included the remaining cases. The following outcomes were examined: death, treatment abandonment, tuberculosis recurrence, and drug resistance.

**Results:**

A total of 14,221 cases reported in 2019 and 2021 were studied; 11,322 lived in areas of high social vulnerability, 2,753 were HIV-infected, and 1,388 were homeless (the total exceeded 14,221, given that there were individuals with more than one vulnerability). All groups had higher incidence, prevalence, and mortality rates, as well as a higher risk of unfavorable outcomes. The risks were highest for homeless individuals, followed by HIV-infected individuals and those living in areas of high social vulnerability.

**Conclusions:**

Compared to individuals without the analyzed vulnerabilities, we found that unfavorable tuberculosis treatment outcomes were associated with different types of vulnerabilities, reflecting individual, social, and program vulnerabilities. Although the individuals studied had access to treatment, several vulnerabilities impacted the likelihood of successful treatment and challenged the tuberculosis control.

## INTRODUCTION

The concept of vulnerability was subjected to an expanded and theoretically consistent formulation with the AIDS epidemic, using several dimensions to comprehensively understand the process of the epidemic, the epidemiological situation, and the health practices required to meet the challenge^1^ ^,^ ^2^. This concept draws attention to a range of individual, collective, and contextual factors that lead to higher susceptibility to infection and determines the resources required by individuals and groups to face the disease^1^ ^,^ ^2^.

The concept of vulnerability encompasses three dimensions of the health-disease production process: individual characteristics, social processes, and aspects of the program, which together affect both the epidemiological profile and outcomes of the disease.

In the present article, the concept of vulnerability, rather than the social determinants of health and social inequalities, was chosen to identify groups who, due to their individual and social conditions, are at a higher risk of acquiring tuberculosis. Moreover, owing to these same characteristics, it may also be more difficult for them to access and use the resources offered by the Tuberculosis Control Program, and they may therefore have less favorable outcomes.

Since the 19th century, there has been a wealth of published evidence linking the incidence of tuberculosis to social determinants of health. However, what we sought to highlight in this study was the persistence of the problem over the centuries, notwithstanding advances in scientific knowledge of the etiologic agent, pathophysiology, clinical manifestations, treatment, and epidemiological behavior of tuberculosis over time in different parts of the world.

São Paulo, the largest city in Brazil in terms of population and one of the richest cities, has a Gross Domestic Product (GDP) higher than that of many countries and is home to social inequalities that negatively affect tuberculosis control. The incidence of tuberculosis in São Paulo in 2019 was 53.55 cases per 100,000 inhabitants, and the mortality rate was 2.5 deaths per 100,000 inhabitants. Due to the Covid-19 pandemic in 2020 and 2021, the incidence decreased to 47.55 and 48.86, due to fewer new cases being detected, and the mortality rate increased to 2.8 and 3.0%, respectively, probably because more severe cases were more likely to be reported. In 2022, the incidence returned to its typical pre-pandemic levels, but the mortality rate continued to grow^3^.

The main rationale for conducting this study was to verify how different groups in vulnerable situations, notwithstanding access to tuberculosis diagnosis and treatment, experienced adverse outcomes.

The main objective of this study was to describe the differences in tuberculosis treatment outcomes in vulnerable groups in the city of São Paulo, presenting the epidemiological behavior and sociodemographic, clinical, and program characteristics of each vulnerable group.

## METHODS

This was a descriptive study with secondary data from the reporting and follow-up system of confirmed cases of tuberculosis among residents of the city of São Paulo. The São Paulo State Health Department (SES/SP) designed an online system, Tuberculosis Patient Control System (TBWEB), for reporting and registering health consultations for confirmed tuberculosis cases, clinical information, therapies administered, case investigations, and contact tracing. The TBWEB aims to reduce the duplication of records and ease follow-up for all services. The individuals in charge of the Tuberculosis control program in each service and epidemiological surveillance center have password access to the TBWEB system. To conduct this study, the authors obtained, by request to the SES/SP, an anonymized database with the selected variables for all reported cases managed in the city of São Paulo, in 2019 and 2021.

The data analyzed included tuberculosis cases in which *Mycobacterium tuberculosis* was confirmed to be the etiological agent. Both incident and prevalent cases were included for 2019 and 2021.

Exposure Variable - Social Vulnerability Groups:

- Homeless individuals were identified using a unique and dedicated zip code entered into the TBWEB system. The sample loss was less than 1% (cases without zip codes). The homeless population was 44,372 in 2019 and 37,200 in 2021 according to the UFMG Public Policy Observatory^4^.
- These tests confirmed the presence of HIV-infected individuals. Unknown data and individuals without HIV test results were 2.6% and 0.4%, respectively. The HIV-infected population was obtained by adding HIV infection data to AIDS prevalence cases for the period, resulting in populations of 113,888 and 118,339 in 2019 and 2021, respectively^5^ ^,^ ^6^.
- Levels of social vulnerability by area of residence: to classify the districts of residence by levels of social vulnerability, we used the São Paulo Social Vulnerability Index (SP-SVI) of the Fundação Sistema Estadual de Análise de Dados Estatísticos (SEADE)^7^.

The SP-SVI consists of socioeconomic indicators (per- capita household income, percentage of households with per-capita income up to half the minimum wage, and percentage of literate heads of household) and demographic indicators (mean age of household heads and percentage of female household heads). The SP-SVI provides vulnerability strata ranging from very low vulnerability (group 1) to very high vulnerability (group 6)^7^.

According to the proportional distribution of census sectors by SP-SVI strata, the districts were classified into three groups: low social vulnerability (districts with 80% or more of census sectors showing extremely low or very low social vulnerability and no census sectors with substandard housing, except for Morumbi with 6% of sectors showing this condition in the Paraisópolis favela); medium social vulnerability (districts with 70 to 79% of census sectors showing low social vulnerability but with up to 5% of census sectors presenting substandard housing); high social vulnerability (districts with a majority of census sectors with medium, high or very high vulnerability and more than 5% of census sectors presenting substandard housing, reaching up to 21% in the case of Jardim Ângela). Losses were 0.77% owing to cases with no information on the neighborhood or district of residence.

The estimates of the resident population in each district in 2019 and 2021 were obtained from the Health Information Center (CEInfo) of the São Paulo Municipal Health Department (SMS/SP)^8^. The populations in 2019 and 2021 were 2,359,911 and 2,387,692 in the low-vulnerability stratum; 2,177,046 and 2,202,696 in the medium-vulnerability stratum; and 8,145,125 and 8,241,088 in the high-vulnerability stratum, respectively.

The outcome variables analyzed were death from tuberculosis (underlying cause), death from tuberculosis (other underlying causes in patients with tuberculosis), treatment abandonment (> 30 days without medication), documented resistance of *M. tuberculosis* to any of the drugs used as first-line treatment, and disease relapse (recurrent episodes of tuberculosis after being declared cured). All data were obtained from the TBWEB, and the outcomes were categorized dichotomously (yes/no).

The sociodemographic variables analyzed were sex, race/color, age (children aged 0 14 years; young individuals aged 15 24 years; young adults aged 25 44 years; adults aged 45 64 years; and the elderly aged 65 years or older), schooling, and employment status. Data loss ranged from 0.06% for age to 30.4% for the educational level.

The clinical variables analyzed included the clinical type, initial treatment regimen, hospital admission, laboratory diagnosis of tuberculosis, presence of diabetes mellitus, presence of AIDS, number of comorbidities, and drug susceptibility tests in cases of treatment failure. Missing clinical variables accounted for less than 2.05%.

The program variables and risk behaviors analyzed were the reporting unit, delay in treatment initiation, type of treatment (DOT or self-administered), alcohol consumption, smoking, and illicit drug use.

The main bias of the study design was the inclusion of only diagnosed and treated tuberculosis cases; that is, only individuals with access to health services. 

The incidence, mortality, and prevalence rates were calculated for each population in each vulnerability group. The fatality rates were calculated using incident cases in 2019 and 2021 that died during those years. 

The percentages in each vulnerability group were calculated for sociodemographic, clinical, and program variables and risk behaviors, and the distributions were tested using the chi-square test (homogeneity test)^9^.

To test the association between the outcomes and vulnerability groups (Pearson’s chi-square test), we calculated the odds ratios using the direct method and exact 95% confidence intervals.

Data were processed and analyzed using STATA version 13.0. No imputation method was used to replace missing data^10^.

The study had a descriptive design, using only secondary data without disclosing patient identification (name and address), to comply with the guidelines and regulatory standards for human research. Therefore, the study was exempt from evaluation by the CEP/CONEP system, according to Resolution 510 of the National Health Council, dated April 7, 2016.

## RESULTS

A total of 1,388 cases of tuberculosis among homeless individuals were studied (9.7% of registered or reported cases), of which 470 (33.8%) were HIV-infected and belonged to two vulnerability groups. The total number of HIV-infected patients was 2,753 (16.1%), of whom 470 (17.0%) were homeless, 1,690 (61.5%) lived in areas of high social vulnerability, 344 (12.5%) lived in areas of medium social vulnerability, and 249 (9.0%) lived in areas of low social vulnerability. The remaining cases, not HIV-infected and not homeless, totaled 10,550 (74.2%), with 8,244 (78.1%) living in areas of high social vulnerability, 1,517 (14.4%) in areas of medium social vulnerability, and 789 (7.5%) in areas of low social vulnerability.

The group with high socioeconomic vulnerability comprised 91.4% of all cases analyzed, with 9.7% homeless, 11.8% HIV-infected, and 69.9% living in areas with high socioeconomic vulnerability.

Table 1 shows the incidence, prevalence, and mortality rates per 100,000 inhabitants and fatality rates per 100 cases in 2019 and 2021 according to the vulnerability groups. Mortality rates were calculated for deaths with tuberculosis as the underlying cause (due to tuberculosis) and deaths with tuberculosis as an associated cause (with tuberculosis). Both rates were included because death tends to be attributed to AIDS as an underlying cause in cases with comorbidities (tuberculosis and AIDS).

**TABLE 1:** Incidence, prevalence, fatality, mortality rates by tuberculosis and with tuberculosis according to vulnerability groups, City of São Paulo, 2019 and 2021.

Vulnerability Groups	Year	Incidence Rate	Prevalence Rate	Fatality Rate	Mortality Rate by tuberculosis^a^	Mortality Rate presenting tuberculosis^b^
Homeless	2019	1156.00	1636.00	8.77	133.00	2.25
	2021	1285.00	1780.00	7.53	134.40	10.75
HIV infection	2019	1065.00	1361.00	7.42	79.03	194.90
	2021	784.20	1017.00	7.43	50.70	147.90
High SEV^c^	2019	54.74	63.06	3.16	21.12	26.27
	2021	49.56	56.39	4.45	26.21	25.00
Medium SEV^c^	2019	29.11	33.80	2.17	14.77	21.73
	2021	27.66	30.58	2.40	15.46	18.04
Low SEV^c^	2019	11.76	12.67	2.11	0.24	0.41
	2021	10.23	11.46	2.40	0.32	0.24

**Note**: Incidence, prevalence, mortality by and with tuberculosis per 100 thousand inhabitants and fatality rate per 100 cases. ^a^ Mortality by tuberculosis: tuberculosis is the underlying death cause; ^b^ Mortality from tuberculosis: death due to any other underlying cause in a patient presenting tuberculosis; ^c^ **SEV:** socioeconomic vulnerability group.

The incidence and prevalence rates are very high among the homeless and HIV-infected individuals. According to the socially vulnerable group in the area of residence, a gradient was observed from most to least vulnerable. The prevalence rates were consistently higher than the incidence rates, and the differences were greater in the most vulnerable groups.

Mortality rates due to tuberculosis showed a similar pattern. Regarding mortality due to tuberculosis as an associated cause, the highest rates were observed among HIV-infected patients, of whom 45.4% were diagnosed with AIDS.

Table 2 shows the distribution of cases in each vulnerability group according to the socioeconomic variables. There were statistically significant differences in the profiles of the groups for all parameters . It is worth noting the predominance of male cases in all groups, the concentration of cases in young individuals and adults, and the higher frequency of cases in the elderly in areas of low social vulnerability (mostly black and mixed), except for residents of areas of low vulnerability. There was a marked difference in schooling and the proportion of unemployed/employed individuals.

**TABLE 2:** Distribution of tuberculosis cases according to socioeconomic and demographic variables in vulnerability groups, City of São Paulo, 2019 and 2021.

Vulnerability Groups		Homeless (%)	HIV infection (%)	High SEV Group (%)	Medium SEV Group (%)	Low SEV Group (%)
Socioeconomic and Demographic Variables						
Sex	Male	87.2	71.2	68.5	64.2	63.5
	Female	12.8	28.8	31.5	35.8	36.5
Race / Color	White	29.3	37.5	33.1	42.5	56.2
	Brown	50.0	44.0	49.1	40.1	27.8
	Black	19.8	16.9	16.5	14.5	11.9
	Asian	0.6	1.4	1.0	2.6	3.9
	Indigenous	0.3	0.2	0.3	0.3	0.2
Age Group	Children	0.2	3.2	3.0	3.2	2.3
	Teens	5.8	11.1	18.0	15.7	12.8
	Young Adults	55.0	47.7	44.1	40.6	41.6
	Adults	35.6	30.0	27.8	28.6	29.3
	Elderlies	3.4	7.9	7.1	11.9	14.0
Total years of Schooling	Illiterate/ 1-3 years	14.8	10.7	11.2	7.4	8.0
	4-7 years	44.2	31.0	31.8	24.9	21.4
	8-11 years	35.1	41.7	43.2	45.4	35.1
	12-15 years	4.8	10.2	10.3	13.7	17.9
	>15 years	1.1	6.4	3.5	8.6	17.6
Employment status	Retired	0.8	4.5	5.4	7.7	9.8
	Unemployed	69.4	31.3	27.4	12.2	10.7
	Housewife/Student	0.4	7.8	10.1	9.4	6.1
	Employed	29.4	56.4	57.1	70.7	73.4

**Note:** proportion of the total in each vulnerability group to characterize the profiles. **SEV:** socioeconomic vulnerability.

Table 3 presents the clinical and treatment characteristics of each group. All the differences were statistically significant. In all groups, pulmonary types prevailed, as did treatment with first-line drugs and laboratory confirmation of diagnosis.

**TABLE 3:** Distribution of tuberculosis cases according to clinical and treatment-related variables in vulnerable groups, City of São Paulo, 2019 and 2021.

Clinical variables		Homeless (%)	HIV- infected (%)	High SVE Group (%)	Medium SVE Group (%)	Low SVE Group (%)
**Clinical Presentation**	Pulmonary	94.4	76.8	83.7	77.8	74.0
	Extrapulmonary	2.2	12.8	12.5	17.4	20.8
	Miliary	3.0	7.5	2.8	3.1	4.0
	Meningitis	0.4	2.9	1.0	1.7	1.2
**Initial Treatment**	First Line	85.5	82.9	94.0	94.0	93.9
	Others	14.5	16.0	6.0	6.0	6.0
						
**Hospitalization**	Yes	55.3	53.9	34.5	36.6	37.3
**Laboratory Confirmation**	Yes	97.5	93.9	95.4	94.4	93.7
**Diabetes Mellitus**	Yes	3.6	4.9	8.1	8.9	7.5
**AIDS**	Yes	18.2	49.0	9.6	8.1	11.8
**Number of Comorbidities**	None	70.2	38.1	71.1	68.2	63.4
	One	25.8	51.9	22.8	23.5	28.0
	Two	2.7	7.1	4.5	6.2	6.7
	Three	1.3	2.9	1.6	2.1	1.9
**Sensibility Test Performed**	Yes	39.8	25.5	22.7	16.0	14.8

**Note:** proportion of the total in each vulnerability group to characterize the profiles. **SEV:** socioeconomic vulnerability.

Severe forms (miliary/meningeal) were more frequent in HIV-positive or low-vulnerability groups with AIDS, whereas extrapulmonary forms were more frequent in the medium or low social vulnerability groups. The main differences among vulnerable groups are hospitalization rates, prevalence of AIDS and other comorbidities, and the need for drug susceptibility testing.

Table 4 presents variables related to the Tuberculosis Control Program and risk behaviors among the vulnerable groups. For all groups, the most common reporting unit was the primary care or outpatient service, although, the homeless were more likely to be diagnosed in an emergency or urgent care setting. The delay between diagnosis and treatment initiation was more frequent in this group and was four times less frequent in medium or low socioeconomic vulnerability groups and twice as frequent in HIV-infected individuals. On the other hand, DOT was more prevalent among the homeless and among those living in areas of high or medium socioeconomic vulnerability.

**TABLE 4:** Distribution of cases in vulnerability groups according to programmatic variables and risk behaviors, City of São Paulo, 2019 and 2021.

Program variables and risk behaviors		Homeless (%)	HIV- infected (%)	High SVE Group (%)	Medium SVE Group (%)	Low SVE Group (%)
**Reporting Unit**	Out-Patient Unit	62.8	48.1	67.7	59.3	55.6
	In-Patient Unit	25.2	43.7	26.8	37.7	37.3
	Prison System	0.1	0.1	0.1	0.3	0.9
	Instituto Clemente Ferreira Reference Center	1.1	2.5	1.1	1.2	3.0
	Emergency Room or First-aid Room	10.5	5.2	4.1	1.3	3.0
	Surveillance Unit	0.3	0.4	0.2	0.2	0.2
**Delay in Starting Therapy**	Yes (interval > 30 days)	24.4	12.4	8.3	6.4	6.8
**Type of Therapy**	Direct Observed Therapy (DOT)	80.4	59.9	79.3	71.3	50.5
	Self- administered Treatment	19.6	41.1	20.7	28.7	49.5
**Alcohol Intake**	Yes	51.0	21.3	23.0	15.5	12.2
**Smoking**	Yes	36.7	20.4	25.7	21.8	16.9
**Illicit Drugs**	Yes	51.6	23.5	21.2	12.0	10.6

**Note:** proportion of the total in each vulnerability group to characterize the profiles. **SEV:** socioeconomic vulnerability.

Alcohol consumption, smoking, and illicit drug use were very common among homeless individuals with tuberculosis, but were halved among HIV-positive individuals and in groups with high social vulnerability. 

Table 5 shows the proportions, odds ratios and confidence intervals of the adverse outcomes in each vulnerability group. The homeless group had the highest risk of treatment abandonment, of tuberculosis recurrence, and more than one adverse outcome. The occurrence of drug resistance was not significant owing to the low number of occurrences. 

**TABLE 5:** Rate, Odds Ratio (95%CI) of unfavorable outcomes of tuberculosis treatment in vulnerable groups, City of São Paulo, 2019 and 2021.

Outcome	Homeless	HIV-infected	High SVE Group	Medium SVE Group	Low SVE Group
Treatment Abandonment	41.6%	28.3%	18.7%	14.3%	13.1%
	OR = 4.11	OR = 2.25	OR = 1.43	OR = 0.74	OR = 0.68
	(3.65-4.62)	(2.04-2.48)	(1.27-1.61)	(0.64-0.85)	(0.56-0.82)
Recurrent Disease	13.1%	11.7%	9.2%	6.8%	6.5%
	OR = 1.80	OR = 1.55	OR = 1.40	OR = 0.74	OR = 0.72
	(1.52-2.13)	(1.35-1.78)	(1.19-1.65)	(0.61-0.90)	(0.55-0.93)
Drug Resistance	1.2%	1.5%	0.9%	1.0%	1.3%
	OR = 1.27	OR = 1.88	OR = 0.82	OR = 1.09	OR = 1.35
	(0.73-2.11)	(1.31-2.71)	(0.54-1.26)	(0.63-1.78)	(0.70-2.42)
More than one negative outcome^a^	61.4%	54.3%	34.1%	29.9%	27.5%
	OR = 3.81	OR = 2.70	OR = 1.26	OR = 0.84	OR = 0.75
	(3.39-4.28)	(2.47-2.95)	(1.15-1.37)	(0.75-0.94)	(0.65-0.87)

^a^Including death due to tuberculosis (TB is the underlying cause) and death not due to TB (TB is an associated cause).. **SEV:** socioeconomic vulnerability. Reference category: total rate for the city.

Compared to non-HIV-positive individuals, HIV-positive individuals are at a higher risk of adverse outcomes. The HIV-positive group had a significant risk of resistance to at least one drug in the first-line regimen. 

The high socioeconomic vulnerability group had a higher risk of treatment abandonment, tuberculosis recurrence, and more than one adverse outcome. By contrast, the medium- and low-vulnerability groups had similar risks and relative risk less than one for all outcomes. 

The proportion of cured cases was lower than 50% among homeless and HIV-positive individuals and nearly 70% in groups according to socioeconomic vulnerability.

## DISCUSSION

The main findings were the concentration of incident and prevalent cases of tuberculosis in some vulnerable groups in the city of São Paulo, with excessive risks among the homeless and HIV-positive, and higher risks among the group with high socioeconomic vulnerability. In addition to inequalities in the occurrence of cases and deaths, it is more difficult for individuals in these groups to undergo treatment for tuberculosis, despite access to health services. Delay in treatment initiation, higher incidence of treatment abandonment, tuberculosis recurrence, and resistance to at least one of the drugs in the first-line regimen in the most vulnerable groups are challenges for the Tuberculosis Control Program. This pattern of occurrence has been described in a previous study using the same data source, which included only incident cases^11^.

Mortality rates from tuberculosis as the underlying cause show a clear gradient among the most vulnerable groups, being particularly high among the homeless or HIV-infected groups that are also disadvantaged in terms of sociodemographic indicators (more men, lower levels of schooling, and higher unemployment) and present worse clinical indicators (prevalence of AIDS, higher hospitalization rates, and use of second-line treatment regimens) and exposure to alcohol, tobacco, and illicit drugs. In the HIV-infected group, severe manifestations of tuberculosis, such as miliary tuberculosis and tuberculous meningitis, were also more frequent. Similarly, the homeless and HIV-infected groups showed the worst outcomes, although supervised treatment achieved 80% coverage.

Similar studies, using registry data or other designs, focusing on tuberculosis among the homeless have been conducted over the past 20 years, and they all revealed comparable findings, both in sociodemographic profiles and exposure to alcohol and drugs. The outcomes studied have always indicated an unfavorable situation for this population^11^ ^-^ ^16^.

Studies led by the Centers for CDC and Prevention in the United States have reported higher cure rates and lower treatment abandonment rates than those observed in São Paulo^12^ ^,^ ^13^. Our findings confirm the results of Brazilian studies of cases of tuberculosis reported in the homeless population^14^ ^,^ ^15^. A systematic review of publications from to 2014-2020 in countries across Europe, Asia, and the Americas found results similar to ours, identifying individual and collective risk factors associated with program deficiencies that led to a lower likelihood of cure, higher rates of treatment abandonment, tuberculosis recurrence, treatment failure and death^16^.

The vulnerabilities to HIV infection are similar to those observed in homeless individuals, with the aggravating factor of HIV infection and a higher incidence of AIDS, especially if individuals are not receiving treatment. A study conducted in Minas Gerais on cases of tuberculosis infection associated with HIV showed that nearly 34% of deaths occurred in patients with AIDS. There was a 19% treatment abandonment rate associated with the younger age group and with the consumption of drugs, alcohol, and tobacco, and cure occurred in only 40% of cases. The main differences from our study were a higher proportion of AIDS cases in Minas Gerais (80% vs. 33%), a lower proportion of individuals undergoing treatment (57% vs. 80%), a lower cure rate (40% vs. 47%), and a smaller number of homeless individuals (4% vs. 14%)^17^.

Both HIV infection and the presence of AIDS and other chronic diseases, as well as excessive consumption of alcohol, tobacco, and other illicit drugs, promote the occurrence of tuberculosis by impairing the immune system, making successful treatment more difficult^16^. This group presented with the most severe types of tuberculosis, miliary and meningeal, probably due to altered immune capacity and the greater proportion of cases with delayed treatment initiation, which not only favors disease progression but also increases the risk of transmission.

Tancredi et al^18^ studied cohorts of new AIDS cases in young individuals and adults in the state of São Paulo between 2003 and 2007 to investigate the impact of AIDS infection and tuberculosis on the likelihood of survival after 12 years of follow-up. In the city of São Paulo, survival was lower among co-infected patients (75% without tuberculosis vs. 60% with tuberculosis).

The high social vulnerability group accounted for the majority (77.4%) of the tuberculosis cases studied, of which 17.0% were HIV infected. Between 2006 and 2013, seven of the nine districts with the highest incidence of tuberculosis in São Paulo belonged to this stratum^11^. Sociodemographic characteristics and risk behaviors were similar to those observed in the HIV-infected group. Clinical and program variables were similar in the medium and low social vulnerability groups, except for the delay in starting treatment, which was higher in this group. Regarding adverse outcomes, the risks were greater in this group, although the cases of tuberculosis and HIV infection were proportionally lower (17%) than those in the medium and low socioeconomic vulnerability groups (18.5% and 24%, respectively). Studies conducted in the 1990s and the 2000s in São Paulo, Rio de Janeiro, and New York found that despite the strong association with the occurrence of HIV/AIDS, poverty in residential areas is an independent risk factor for tuberculosis incidence and mortality^19^ ^-^ ^21^. 

In 2018, Maciel et al.^22^, studying incident cases of tuberculosis in the city of Rio de Janeiro between 2008 and 2013, found that the treatment dropout rate was directly related to the concentration of poor and extremely poor individuals in each district, with a lower human development index-education component, worse urban infrastructure conditions, in addition to pointing to urban violence as an obstacle to tuberculosis surveillance by family health teams. 

The medium social vulnerability group had intermediate sociodemographic characteristics between those of the high and low social vulnerability groups. The proportion of cases with tuberculosis and AIDS diagnoses was similar to that in the high social vulnerability group, and there were more extrapulmonary types. Alcohol and drug consumption were lower, and smoking rates were similar. Lower rates of treatment dropout, tuberculosis recurrence, and more than one adverse outcome may be related to more stable social conditions and the possibility of regularly maintaining treatment. In the 2006-2013 period, two of the nine districts with the highest incidence of tuberculosis belonged to this stratum^11^.

In the low social vulnerability group, cases of tuberculosis and HIV infection (24%), a higher frequency of AIDS (11.8%), and 26% of extrapulmonary types of tuberculosis contributed to worse outcomes. This composition of factors may explain why the risk of adverse outcomes was similar to that observed in the previous group. Another reason for the lack of a significant difference in outcomes between the medium and low social vulnerability groups may be the small number of occurrences registered in these two groups.

Both homeless and HIV-positive patients have lower cure rates, as observed in different countries and regions^23^ ^-^ ^30^. Both groups share the effects of precarious living conditions and lower immunity caused by HIV or very high alcohol and drug consumption among homeless individuals. For HIV-positive patients, the more severe clinical forms of tuberculosis make curing more difficult and time-consuming. For the homeless, constant motility interrupts treatment. Frequent hospitalizations indicated a higher frequency of worsening conditions requiring hospital care. The main complications in these groups were treatment abandonment and loss to follow-up. 

This study had some limitations, including the use of secondary data obtained from a health information system designed to register service activities. In general, the variables were adequately completed, but some data were missing. Another limitation is the number of cases in the medium and low socioeconomic vulnerability groups. The main bias of the study design was the inclusion of only diagnosed and treated tuberculosis cases; that is, only individuals with access to health services^31^. Finally, we were unable to work with individuals deprived of their liberty because of the difficulty in relating this group to the general population. Some people detained in the municipality do not live in São Paulo and may have come from other states.

Conversely, incident and prevalent cases were included, increasing the possibility of fully observing outcomes throughout the follow-up period. The main advantage of this study is that it described aspects related to each vulnerability group, highlighting the epidemiological profile, sociodemographic and clinical characteristics, risk behaviors, and aspects of the program.

**Conclusion:** By characterizing the most vulnerable groups responsible for a considerable proportion of disease occurrence, our findings may be useful for the Tuberculosis Control Program. Programme interventions must consider social inequalities and vulnerabilities when tuberculosis is concentrated in certain subgroups of a population with accumulated disadvantages, making prevention and successful treatment more challenging.

## Data Availability

Research data is only available upon request.
